# Substrate-mediated oxidative stress alleviation and rhizosphere microbiome modulation enable submerged macrophyte restoration in high-turbidity water

**DOI:** 10.3389/fpls.2026.1961849

**Published:** 2026-10-30

**Authors:** Taofeng Lu, Yunhua Huang, Ziwei Liu, Yanjun Wu, Suqing Wu, Chunzhen Fan

**Affiliations:** 1 Guizhou University of Traditional Chinese Medicine, Guiyang, China; 2 College of Life and Environmental Science, Wenzhou University, Wenzhou, Zhejiang, China; 3 National and Local Joint Engineering Research Center of Ecological Treatment Technology for Urban Water Pollution, Wenzhou University, Wenzhou, Zhejiang, China

**Keywords:** antioxidant defense system, oxidative stress alleviation, rhizosphere microbial community, suspended planting substrate, *Vallisneria spinulosa* Yan, water purification

## Abstract

**Introduction:**

Ecological restoration of submerged macrophytes in highly turbid waters is severely hampered by light attenuation, which imposes significant oxidative stress on plants and disrupts their physiological homeostasis. While suspended planting technology offers a solution by optimizing light exposure, the critical role of planting substrates in modulating plant stress tolerance and rhizosphere resilience remains largely unexplored—a knowledge gap that this study addresses for the first time. This study is novel in systematically linking substrate physical properties to plant redox regulation and rhizosphere microbiome assembly under turbid-water stress, providing an integrated plant–microbe perspective previously unaddressed.

**Methods:**

In this study, we cultivated *Vallisneria spinulosa* Yan using four suspended substrates (plastic mesh, palm mat, linen cloth, and bamboo woven strips) in outdoor flume experiments, aiming to evaluate their effects on plant physiology, water remediation, and associated microbial networks.

**Results:**

Our results demonstrated that the linen cloth substrate most effectively alleviated oxidative damage, as evidenced by significantly enhanced root development, biomass accumulation, and leaf photosynthetic pigment content, alongside a marked reduction in physiological oxidative stress markers. Functionally, all treatments achieved over 90% turbidity removal; however, the linen cloth group exhibited superior purification efficiency, with the highest total nitrogen and total phosphorus removal loads (2.32 × 10^-4^ and 2.70 × 10^-4^ g·m^-2^·d^-1^, respectively). Critically, microbial community analysis revealed that the linen cloth substrate fostered a more complex and interconnected rhizosphere network, with a notable enrichment of a denitrifying bacterial family (*Comamonadaceae*), indicating a synergistic enhancement of plant–microbe mutualism under stress.

**Discussion:**

Collectively, our findings identify linen cloth as the optimal substrate for suspended planting, not merely as a physical support, but as a catalyst for reinforcing the plant’s antioxidant defense system and orchestrating a beneficial rhizosphere microbiome, thereby providing a robust theoretical framework for applying this redox-based ecological engineering strategy in turbid water restoration.

## 1 Introduction

Surface water is an indispensable resource for maintaining ecological balance and biodiversity (Syeed et al., 2023). However, rapid urbanization, agricultural runoff, industrial discharge, and natural processes have caused severe pollution, with elevated turbidity emerging as a globally concerning issue (Yan et al., 2023). High turbidity not only impairs water aesthetics and disrupts aquatic food webs but also induces physiological stress to submerged macrophytes by reducing light penetration (Chang et al., 2024). Insufficient light limits photosynthetic electron transport, leading to the overproduction of reactive oxygen species (ROS) in plant cells. Excessive ROS attack membrane lipids, proteins, and nucleic acids, causing oxidative damage and compromising plant establishment and survival (Nagayama and Tonooka, 2023; Wang et al., 2015). Simultaneously, suspended particles act as carriers of nutrients and pollutants, exacerbating eutrophication and further intensifying the stress regime (Lizotte and Locke, 2018).

Current remediation techniques for turbid waters include flocculation, microbial inoculation, constructed wetlands, and phytoremediation. Flocculation, though operationally simple, generates large sludge volumes and risks secondary pollution; microbial approaches often fail due to competition with indigenous communities and hydrological disturbances; constructed wetlands are effective but prone to clogging by suspended solids (Asadi-Ghalhari et al., 2023; Huang F. et al., 2022; Feng et al., 2022). In contrast, submerged macrophyte-based restoration—called “underwater forest” approach—offers environmentally friendly, cost-effective, and long-lasting benefits (Chen et al., 2022; Chao et al., 2021). As primary producers, submerged plants absorb nitrogen and phosphorus, degrade organic pollutants, and stabilize sediments, thereby enhancing water clarity (Feng et al., 2022; Chen et al., 2022; Yu et al., 2025). *Vallisneria* spp., characterized by low light compensation points, are widely used as pioneer species for ecological restoration (Lowden, 1982; Wang et al., 2024; Gao et al., 2017).

Nevertheless, direct *in situ* planting in turbid waters is hindered by light limitation and sediment contamination, resulting in poor establishment and chronic oxidative stress. Auxiliary techniques such as underwater light supplementation, compartmented planting, and floating beds have been applied, but each has inherent drawbacks (high energy consumption, habitat fragmentation, or limited water-column improvement) (Wang et al., 2020; Zhu et al., 2022). Suspended planting technology, which adjusts the planting plane to an optimal water depth, can effectively reduce light inhibition for submerged plants in high-turbidity water. Thus, it has shown promise for turbid-water restoration. The planting substrate is a critical component of this technology**—**not merely as a physical support, but as an interface that modulates root development, oxygen availability, and microbial colonization. An ideal substrate should possess sufficient porosity to maintain aerobic conditions, facilitate nutrient exchange, and provide niches for beneficial microorganisms (Wang et al., 2022; Cui et al., 2018; Jiang et al., 2010; Liang et al., 2025).

To date, most studies have focused on conventional sediment-based planting and have paid limited attention to how substrate properties influence plant oxidative stress responses and rhizosphere microbial community assembly in suspended systems. In particular, the linkages among substrate type, plant antioxidant defense systems, and microbial network resilience remain largely unexplored. Therefore, this study targeted *Vallisneria spinulosa* Yan (*V. spinulosa* Yan) as a model species and selected four substrates—plastic mesh, palm mat, linen cloth, and bamboo woven strips—to investigate their effects on plant growth physiology, oxidative stress markers, water purification efficiency, and rhizosphere microbial community structure under high-turbidity conditions. We hypothesized that substrates with favorable physical structures would alleviate oxidative damage, enhance antioxidant enzyme activities, and promote the enrichment of functional microorganisms, thereby improving the overall resilience of the plant–microbe symbiotic system. Our objective was to identify the optimal substrate and to provide a mechanistic framework for the engineering application of suspended planting technology in turbid waters, with a focus on redox regulation and rhizosphere ecology.

## 2 Materials and methods

### 2.1 Experimental setup and water preparation

The experiment was conducted at the outdoor experimental platform of Wenzhou University (120°69′E, 27°92′N, Zhejiang, China). Four PVC barrels (top diameter 101 cm, bottom diameter 84 cm, height 84 cm; surface area 0.785 m², effective volume 500 L) were used as experimental units, which was suitable for long-term outdoor water-holding experiments without leakage due to its dense, weather-resistant, and impermeable properties. As shown in **Supplementary Figure 1**, a horizontal suspension rod with a hand-cranked winch was fixed above each barrel to adjust the planting plane to a 20-cm depth below the water surface. The 20-cm planting depth was selected based on the light compensation depth of *V. spinulosa* reported in previous studies. Preliminary trials confirmed that this depth supported stable photosynthetic performance and biomass accumulation in *V. spinulosa* under comparable turbidity levels. Each substrate was layered to form a planting mat with adjustable weights to the four corners of the mat frame and suspended by nylon ropes attached to the horizontal rod, ensuring that the mat remained fully submerged at the designated depth regardless of material buoyancy. The counterweights (stainless steel washers, 50 g each) were pre-calibrated to offset the buoyancy of each substrate type, and the suspension ropes were tensioned to prevent vertical displacement under minor water disturbances. This configuration stably submerged and maintained the planting plane at a specific water depth. All barrels were placed under natural sunlight to simulate real-world light conditions. The experiment was conducted from July to August 2024, during which the mean daily water temperature ranged from 29.1 to 33.9 °C. The barrels were positioned in an open area without shading, and all barrels received identical natural illumination.

The experimental water was prepared according to China’s Class V surface water quality standards (GB 3838-2002). Analytical-grade KH_2_PO_4_, NH_4_Cl, and KNO_3_ were used as nutrient sources. To simulate turbid water, sediment from Wenrui Pond River was spread in thin layers (approximately 2 cm depth) and dried under shade at ambient temperature for one week, with periodic turning every 24 h to ensure uniform drying, ground using a crusher, sieved (60-mesh), and mixed into the water. After standing for two days, the supernatant turbidity was measured with a HACH 2100Q portable turbidimeter and adjusted to exceed 90 NTU through stepwise addition of the pre-sieved sediment (approximately 0.5–1.0 g per addition) with continuous stirring, followed by a 30-min stabilization period before each re-measurement. This procedure was iterated until the turbidity fell within 90–110 NTU. Prior to introducing plants, the final turbidity of each barrel was confirmed by triplicate measurements. The influent water for all four barrels was pumped simultaneously from the same 2000 L influent tank using a submersible pump with a four-way splitter, ensuring identical initial water quality across all treatments. Barrels were filled once at the start of the experiment (day 0) with no further water exchange during the 42-day experimental period, except for evaporation compensation and sampling. Initial water quality parameters are listed in **Table 1**.

**Table 1:** Experimental influent water quality.

Water quality indicators	TN	TP	NH_4_^+^-N	NO_3_^-^-N	PO_4_³^-^-P
Concentration(mg·L^-^¹)	2~3.5	0.2~0.3	0.9~1.2	1.2~1.8	0.16~0.35

### 2.2 Plant material and acclimation

*V. spinulosa* Yan individuals were purchased from Honghu Xianghe Trading Co., Ltd. (Hubei, China). Healthy, uniform-sized plants (6–8 leaves, length 15 ± 5 cm, without ramets) were selected and pre-cultured for 10 days in clean water at 20–25 °C to acclimate to the experimental environment. During acclimation, plants were maintained in 200 L plastic tanks filled with dechlorinated tap water under natural light. The water was renewed every 3 days to maintain water quality, and any dead or senescent leaves were removed daily. Only plants that remained healthy (green leaves, no visible necrosis, chlorosis, or mechanical damage) after the 10-day acclimation period were selected for the formal experiment. The plants were obtained from a commercial nursery to ensure genetic and developmental uniformity across all experimental treatments, thereby minimizing confounding effects arising from field-collected individuals with potentially different age structures, health statuses, or stress exposure histories. This standardization was critical for attributing observed differences specifically to substrate type.

### 2.3 Experimental design

Due to spatial constraints, a single mesocosm unit (one barrel) was established for each substrate treatment. In this experimental design, the barrel is the experimental unit, and each barrel contained 100 plants as subsamples. Thus, the true replication at the treatment level is n = 1 per substrate. To ensure data robustness and minimize measurement bias, three independent subsamples (technical replicates) were collected from each unit at every sampling event for all physiological, water quality, and microbial analyses. All results are presented as means ± standard deviations of these technical replicates. Because treatment-level replication was not achieved, all data are presented as descriptive comparisons among the four substrate treatments. Statistical inferential tests (e.g., ANOVA) were performed with the understanding that the subsamples are pseudo replicates; therefore, the reported p-values are interpreted as descriptive measures of within-treatment variability rather than as definitive evidence of treatment effects. Readers should interpret the differences among treatments with this limitation in mind. A single-factor design was adopted with four substrate treatments: plastic mesh (M1), palm mat (M2), linen cloth (M3), and bamboo woven strips (M4). Each substrate was layered to a thickness of 2 cm, providing a planting area of 0.28 m² per barrel. One hundred *V. spinulosa* Yan individuals were planted per barrel. The plants were evenly distributed across the substrate surface at a spacing of approximately 5 cm between adjacent individuals. For M3 (linen cloth) and M2 (palm mat), roots were gently inserted into the fiber interstices to ensure initial physical contact; for M1 (plastic mesh), roots were threaded through the mesh openings; for M4 (bamboo strips), roots were placed between adjacent bamboo slats. The planting plane was suspended at 20 cm depth, a depth chosen to balance adequate light availability for photosynthesis against the risk of photoinhibition at the water surface under turbid conditions, and the experiment lasted for 6 cycles (7 days each), with continuous monitoring. Each 7-day cycle represented one complete hydraulic retention period without external water exchange. Water lost due to evaporation was replenished daily with deionized water to maintain a constant water volume of 500 L per barrel; the volume of deionized water added was recorded daily.

### 2.4 Plant growth and physiological measurements

At the end of the experiment on day 42, leaf length, width, perimeter, and area were measured using a Wansheng Root Analyzer (LA-S series, China). For each treatment, 20 randomly selected individuals (approximately 20% of the total 100 plants per barrel) were used for morphological measurements. Roots were rinsed with distilled water, and root length, diameter, surface area, and volume were quantified using the LA-S Plant Image Analysis System. For biomass, shoots and roots were separated, dried at 80 °C to constant weight, and weighed as aboveground and underground biomass (total = sum).

For photosynthetic pigments, 0.2 g fresh leaf tissue from the middle section of mature leaves (avoiding leaf tips and bases) was extracted in 10 mL of 95% ethanol in darkness at room temperature (25 °C) in sealed glass tubes for 24 h. The tubes were gently shaken every 6 h to ensure complete extraction. Absorbance at 665, 649, and 470 nm was measured with a UV-Vis spectrophotometer against a 95% ethanol blank to calculate chlorophyll a, chlorophyll b, carotenoids, and total chlorophyll. Three replicate extractions were performed per barrel (subsamples).

For antioxidant enzyme activities and oxidative stress indicators, fresh leaf samples (approximately 0.5 g fresh weight) were homogenized in 5 mL of pre-chilled extraction buffer (50 mM phosphate buffer, pH 7.0) using a mortar and pestle kept on ice. The resulting homogenate was centrifuged at 10,000 rpm for 15 min under refrigeration (4 °C), and the clear supernatant was carefully recovered for subsequent enzyme assays. All extraction procedures were conducted at low temperature (ice-bath), and spectrophotometric measurements were completed within 6 h of tissue collection to minimize enzyme degradation. Superoxide dismutase (SOD) activity was determined by the nitroblue tetrazolium photoreduction method; peroxidase (POD) activity was measured using guaiacol as substrate; catalase (CAT) activity was assessed by monitoring H_2_O_2_ decomposition at 240 nm. Malondialdehyde (MDA) content, a marker of lipid peroxidation, was measured via the thiobarbituric acid reactive substances (TBARS) assay. SOD activity was expressed as U g^-^¹ fresh weight (one unit defined as the amount of enzyme causing 50% inhibition of NBT photoreduction at 560 nm); POD and CAT activities were expressed as U g^-^¹ fresh weight min^-^¹ (one unit defined as a 0.01 absorbance change per minute at 470 nm for POD and a 0.01 absorbance change per minute at 240 nm for CAT). All enzyme activities were expressed on a fresh weight basis. All assays were performed in triplicate per barrel (three independent subsamples from each barrel).

### 2.5 Water quality analysis

Water samples were collected at each cycle (every 7 days) from the mid-water column at approximately 40 cm below the water surface using a 1 L water sampler and additionally at 12-h intervals during the first cycle for turbidity kinetics. Total nitrogen (TN) was determined by alkaline potassium persulfate digestion-UV spectrophotometry; total phosphorus (TP) by molybdenum-ammonium spectrophotometry; nitrate-N (NO_3_^-^-N) by UV spectrophotometry; ammonia-N (NH_4_^+^-N) by Nessler’s reagent method; and phosphate (PO_4_³^-^-P) by the molybdenum-antimony method. pH, dissolved oxygen (DO), water temperature (WT), and oxidation-reduction potential (ORP) were measured *in situ* immediately after collection with a Hach portable electrochemical analyzer. For nutrient analyses (TN, TP, NH_4_^+^-N, NO_3_^-^-N, PO_4_³^-^-P), water samples were filtered through 0.45-μm glass fiber filters prior to analysis and stored at 4 °C in the dark; all nutrient determinations were completed within 24 h of sampling. Turbidity was recorded with a HACH 2100Q turbidimeter (USA). Suspended particle size distribution was determined using a Mastersizer 3000 laser diffraction analyzer (UK), both for water column particles directly and for particles attached to leaves. For leaf-attached particles, 5 g of fresh leaf tissue was sonicated in 50 mL distilled water at 40 kHz for 5 min, vortex-mixed for 2 min, and centrifuged at 500 × g for 5 min to remove large debris; the supernatant containing detached particles was then analyzed using the Mastersizer 3000. All water quality analyses were performed in triplicate per barrel (three independent subsamples per sampling event).

### 2.6 Scanning electron microscopy

Substrate samples (before and after the experiment) and leaf samples were fixed, dehydrated, and examined with a scanning electron microscope to observe surface microstructure and particle adhesion. Leaf samples were fixed with glutaraldehyde, dehydrated in graded ethanol, and then subjected to SEM observation. Substrate samples were collected before experiment setup (day 0) and after experiment completion (day 42), cut into 5 mm × 5 mm pieces, fixed with 2.5% glutaraldehyde in 0.1 M phosphate buffer (pH 7.2) for 4 h at 4 °C, rinsed three times with the same buffer (10 min each), dehydrated through a graded ethanol series (30%, 50%, 70%, 80%, 90%, 95%, 100%, 15 min per step), critical-point dried using liquid CO_2_ as the transitional fluid, sputter-coated with gold at 15 mA for 60 s, and observed using a scanning electron microscope (ZEISS Sigma 300, Germany) at an accelerating voltage of 5 kV at 200 μm and 20 μm magnifications.

### 2.7 Rhizosphere and surface microbial community analysis

Microbial samples were collected from four compartments: leaf surfaces (L), root surfaces (R), substrate surfaces (S), and the water column (W) of each treatment, at two time points (mid-stage: day 21; late-stage: day 42). Sample codes were defined as follows: L, R, S, and W denote leaf, root, substrate, and water compartments, respectively; numbers 1–4 denote M1–M4 substrate groups; A and B denote mid-stage and late-stage sampling, respectively. Combinations such as LA, LB, RA, RB, SA, SB, WA, and WB thus represent specific sample types at specific time points; when substrate differentiation is needed, the group number is appended (e.g., LA3 = M3 mid-stage leaf). See **Supplementary Table 1** for the full sample-code mapping.

Leaf samples were rinsed with sterile phosphate-buffered saline (PBS), then microbial cells were detached by ultrasonication (5 min, 40 kHz) and vortex mixing, followed by collection on 0.22 μm membranes. Root and substrate samples were washed with PBS buffer, and surface microbes were recovered by ultrasonication and buffer washing, then filtered onto 0.22 μm membranes. Water samples (500 mL) were directly filtered through 0.22 μm membranes. All membrane filters were preserved at –80 °C until DNA extraction. Samples were sent to Shanghai Meiji Technology Co., Ltd. (Shanghai, China) for bacterial 16S rRNA high-throughput sequencing. Total genomic DNA was extracted from the membrane filters using the PowerSoil DNA Isolation Kit (MoBio Laboratories, Carlsbad, CA, USA) following the manufacturer’s protocol. DNA quality and concentration were assessed by 1% agarose gel electrophoresis and spectrophotometry (NanoDrop 2000, Thermo Scientific). The V3–V4 region was amplified with primers 338F/806R using the following thermal cycling conditions: an initial heating step at 98 °C for 1 min; then 30 amplification cycles consisting of denaturation at 98 °C for 10 s, primer annealing at 50 °C for 30 s, and strand extension at 72 °C for 30 s; followed by a final extension at 72 °C for 5 min. Each 25-μL reaction contained 12.5 μL of 2× Phusion High-Fidelity PCR Master Mix, 1 μL each of forward and reverse primers (10 μM stock), and approximately 20 ng of template DNA. PCR products were purified using a Gel Extraction Kit (Qiagen, Germany) and quantified using a Qubit 2.0 fluorometer. Raw sequences were quality-filtered, with low-quality reads (average quality score < 20 or length < 200 bp) removed, and clustered into OTUs at 97% similarity, using the UPARSE pipeline. Chimeric sequences were identified and removed using UCHIME. Taxonomy was assigned against the SILVA database v138. To infer the functional potential of the microbial communities, PICRUSt2 (Phylogenetic Investigation of Communities by Reconstruction of Unobserved States 2) was applied to the OTU representative sequences (Douglas et al., 2020). Briefly, the OTU abundance table was first normalized by predicting 16S rRNA copy numbers for each OTU using the castor hidden-state prediction algorithm. The normalized OTU table was then used to predict gene family abundances, specifically Kyoto Encyclopedia of Genes and Genomes (KEGG) Ortholog (KO) abundances and Enzyme Commission (EC) numbers, based on the reference genome database integrated in PICRUSt2. Functional predictions were performed using the default parameters of the PICRUSt2 pipeline, and the resulting KO abundances were collapsed to KEGG pathway-level annotations for downstream comparative analysis among substrate treatments. Alpha diversity (Shannon, Simpson, Ace, Chao) and beta diversity (Bray–Curtis) were calculated. Partial least squares discriminant analysis (PLS-DA) was performed at the genus level to discriminate community differences among substrates and compartments. Differential abundance analysis identified taxa with the highest mean relative abundance differences among substrate types.

### 2.8 Statistical analysis

All data are expressed as mean ± standard deviation calculated from the three technical replicates (subsamples) per sampling event from each barrel. Given the exploratory nature of this study and the lack of treatment-level replication (n = 1 per substrate), the reported p-values from ANOVA should be interpreted cautiously—they reflect within-barrel variability rather than serving as definitive evidence of true treatment effects. The numerical comparisons among treatments are presented descriptively, and the observed differences are discussed in the context of consistent trends across multiple independent indicators. One-way ANOVA with Tukey’s HSD post-hoc test was used as an exploratory tool for multiple comparisons among the four substrate treatments. However, we emphasize that these statistical results are descriptive, not inferential, due to the absence of independent biological replicates at the barrel level. All statistical outputs are provided as supplementary information for reference. Significance was set at p < 0.05. Analyses were performed using R software (v.4.2.1) with packages vegan, ggplot2, mixOmics, and stats.

## 3 Results

### 3.1 Plant growth and oxidative stress responses

All data presented below were obtained from three technical replicates per sampling event from each treatment unit, and are expressed as mean ± SD. The consistent trends observed across multiple parameters provide internal validation of the measured differences among treatments.

#### 3.1.1 Growth performance

Biomass allocation and root-shoot traits showed notable differences among the four substrate treatments. The underground biomass showed relatively small differences among the four treatments, whereas aboveground and total biomass exhibited more pronounced differences. As shown in **Figure 1A**, the aboveground biomass proportion ranged from 62% to 71%, with M3 (linen cloth) having the highest (71%). **Figure 1B** shows that M3 achieved the highest underground (0.07 g), aboveground (0.18 g), and total (0.25 g) biomass—the aboveground biomass of M3 was 2.38-fold that of M2, consistent with superior shoot development in this treatment.

**Figure 1:**
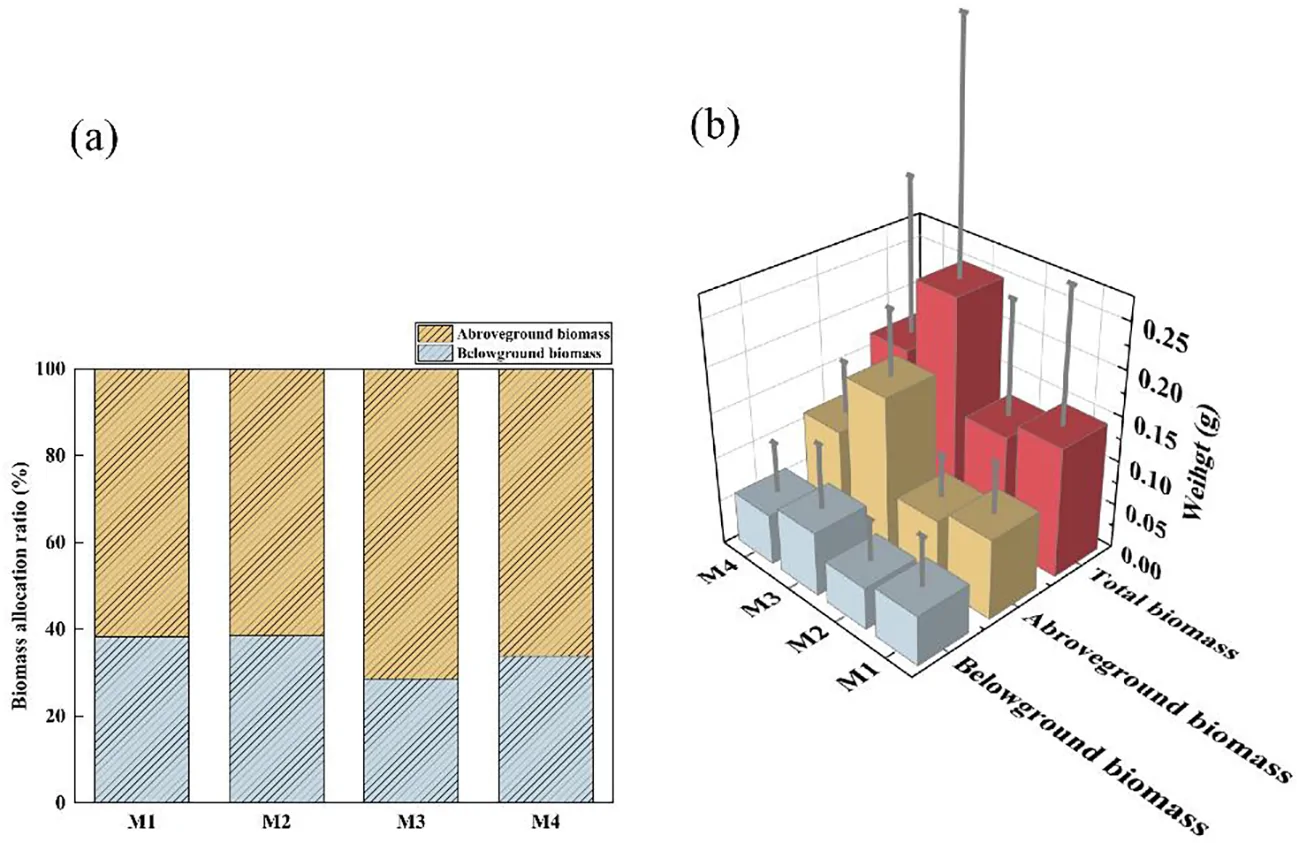
Proportion of *V. spinulosa* Yan biomass **(A)** and differences in biomass **(B)** among different planting substrate groups.

Leaf and root growth indicators (**Figure 2**) further reflected the advantage of M3. M3 exhibited the greatest root length, root surface area, root volume, and root diameter, as well as the longest leaf length (38.41 cm), leaf area (26.71 cm²), and leaf perimeter (77.73 cm). In contrast, M2 (palm mat) showed the poorest performance, with root length less than one-third of M3 and root volume only 0.52 cm³. M1 (plastic mesh) had weak root establishment due to large mesh openings, while M4 (bamboo) performed intermediately. These results suggest that linen cloth provides a favorable physical environment for root anchorage and expansion, which was associated with vigorous shoot growth.

**Figure 2:**
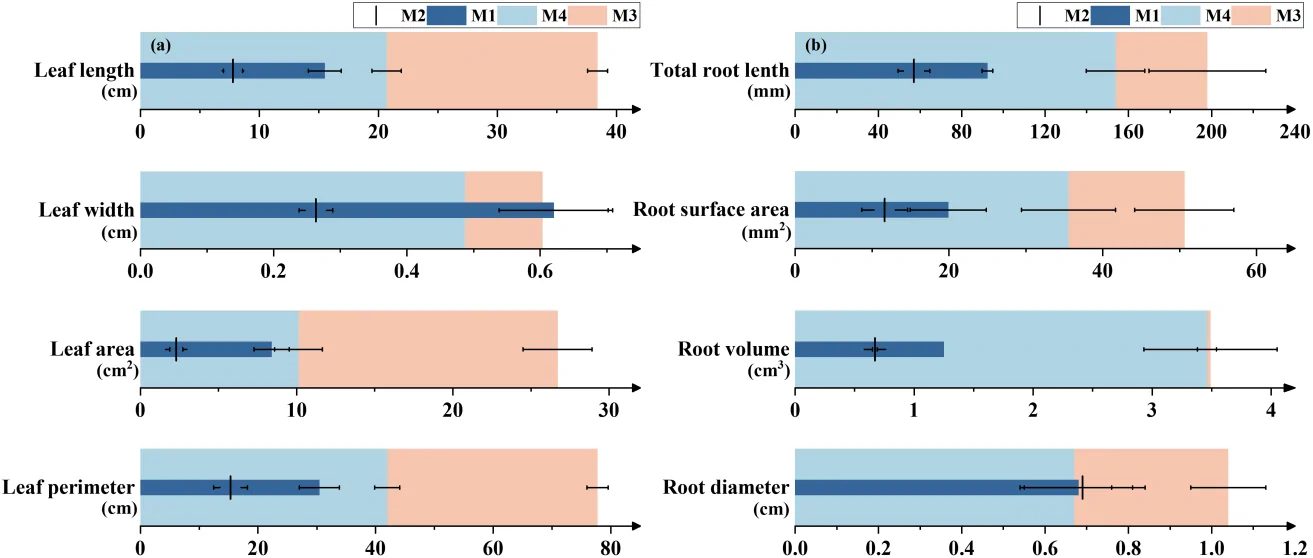
Leaf **(A)** and root growth indicators **(B)** of *V. spinulosa* Yan under different planting substrates.

#### 3.1.2 Photosynthetic pigments and antioxidant physiology

Photosynthetic pigment contents are shown in **Figure 3**. M3 consistently had the highest chlorophyll a (0.83 mg/g), chlorophyll b (0.36 mg/g), carotenoids (0.18 mg/g), and total chlorophyll (1.19 mg/g), suggesting enhanced light-harvesting capacity under turbid conditions. This is likely due to the well-developed root system of M3 plants, which facilitates nutrient uptake for pigment synthesis.

**Figure 3:**
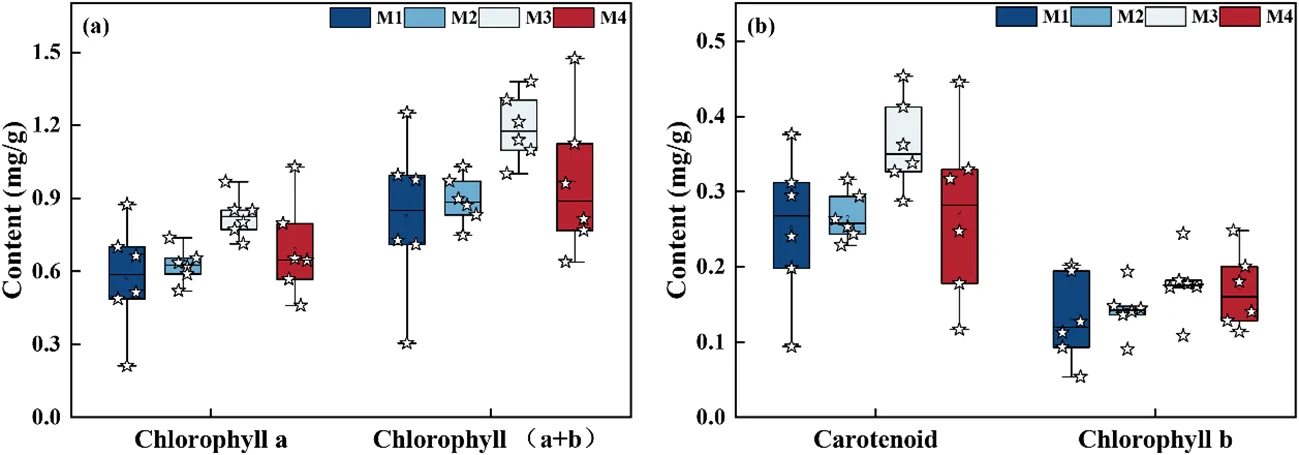
Photosynthetic pigments of *V. spinulosa* Yan under different planting substrates: **(a)** chlorophyll a and chlorophyll (a + b), **(b)** carotenoids and chlorophyll b.

Crucially, oxidative stress markers and antioxidant enzyme activities showed marked differences among the substrate treatment (**Figure 4**). SOD activity (**Figure 4A**) increased overtime in all groups, but M3 showed the most gradual and sustained increase, reaching 476.65 U/g on day 14, whereas M1 exhibited a sharper rise later, indicating higher stress levels. CAT activity (**Figure 4C**) peaked on day 28 in M2 but declined thereafter; by day 42, M3 had the highest CAT (19.71 U/g). POD activity (**Figure 4D**) increased with time in all except M1, with M3 showing a steady upward trend. MDA content (**Figure 4B**), an index of lipid peroxidation, continuously rose in M1, while other groups showed an initial increase followed by a decrease. M3 maintained the lowest MDA throughout the experiment, suggesting that M3 sustained relatively minimal oxidative damage to cell membranes.

**Figure 4:**
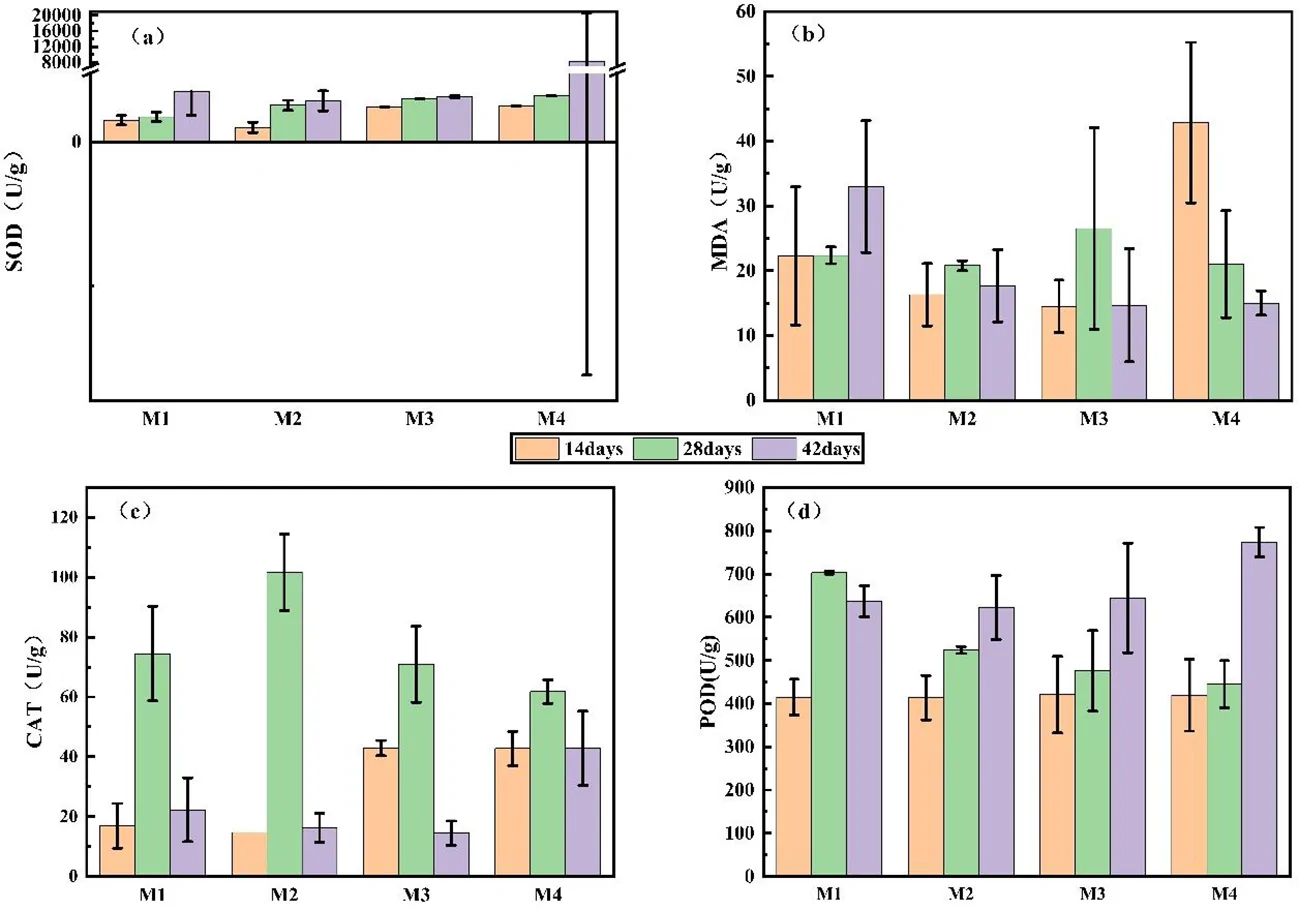
Activities of SOD **(A)**, CAT **(C)**, POD **(D)** and the content of MDA **(B)** in *V. spinulosa* Yan under different planting substrates.

Overall, these physiological data suggest that the linen cloth substrate was most effective among the tested treatments in alleviating oxidative stress in *V. spinulosa* Yan, as reflected by lower MDA levels and higher, more stable SOD, POD, and CAT activities. This coordinated antioxidant defense is consistent with improved maintenance of redox homeostasis under high-turbidity stress.

### 3.2 Water purification performance

#### 3.2.1 Turbidity removal and particle size dynamics

All four treatments achieved >90% turbidity removal within 7 days (**Figure 5A**), with the fastest reduction occurring in the first 24 hours (66.7–82.5% removal). Nonlinear fitting of turbidity decline (y = a·x^b^) showed that M3 had the largest absolute b value (**Figure 5B**), suggesting the fastest turbidity reduction rate among the four treatments. This difference may be partly attributed to the high specific surface area and rough fiber structure of the linen cloth, which may enhance physical adsorption.

**Figure 5:**
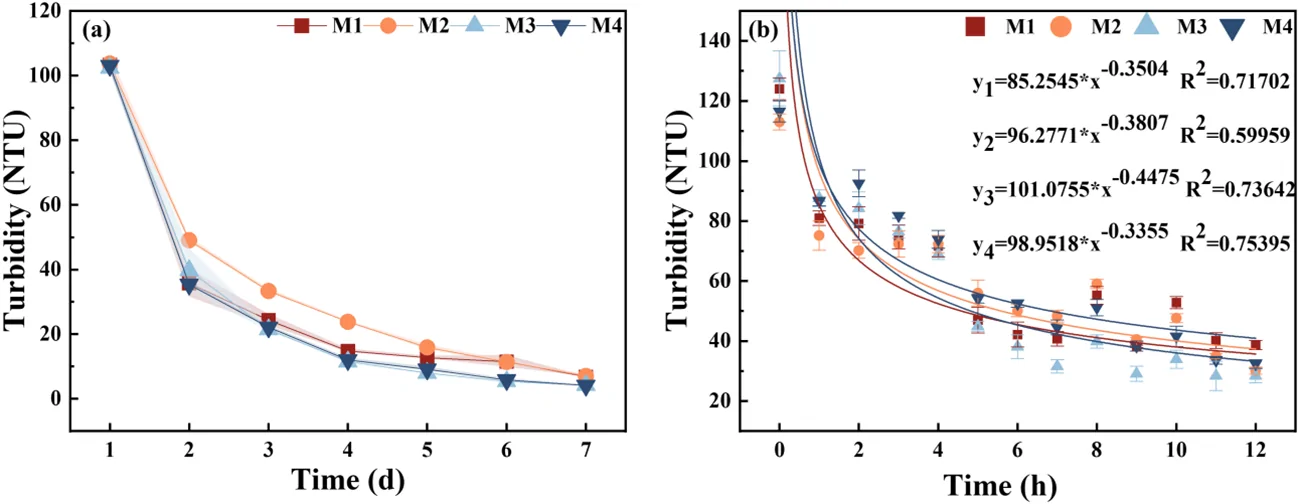
Changes in water turbidity under different planting substrates: **(A)** 7-day variation in water turbidity; **(B)** 12-hour variation in water turbidity.

Particle size distribution (**Figure 6**) shifted markedly over time. Initially, 70–77% of suspended particles were <5 μm. By day 7, the proportion of <5 μm particles decreased to 20–42%, while particles >250 μm increased up to 57%, with M3 showing the most obvious shift. This suggests bioflocculation—the aggregation of fine particles into larger flocs—mediated by extracellular polymeric substances from biofilms (Flemming and Wingender, 2010). Consistently, particles attached to leaves (**Supplementary Figure 2**) were predominantly small (1–5 μm and 5–10 μm), and M3 leaves had no particles >250 μm, which may indicate that larger flocs in the M3 water column were less firmly attached and more prone to sedimentation.

**Figure 6:**
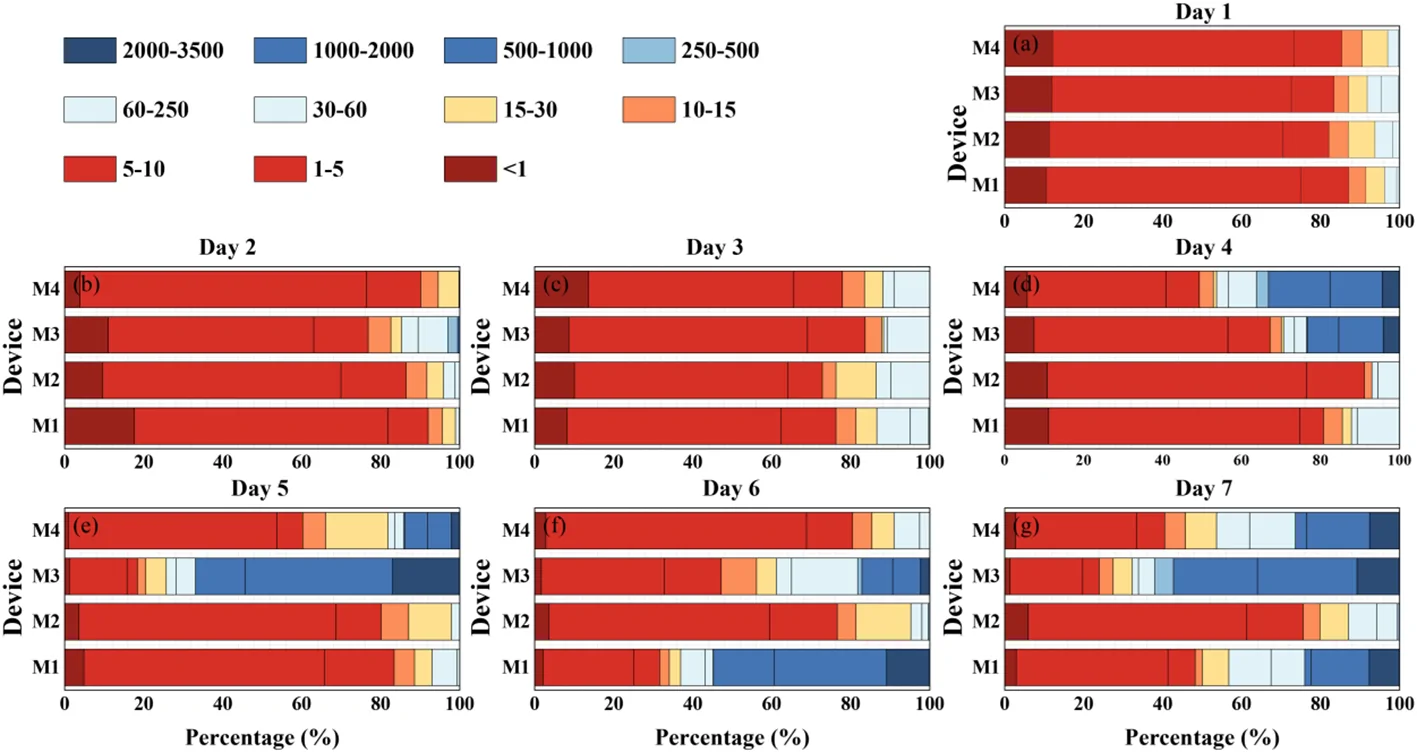
Changes in suspended particle size of water bodies during an influent cycle under different planting substrates.

#### 3.2.2 Nutrients removal

DO was notably higher in M3 (~7 mg·L^-^¹) than in other groups (**Figure 7B**), which may partly reflect stronger photosynthetic activity in the M3 treatment, although other factors such as temperature and reaeration cannot be excluded. pH (**Figure 7C**), WT (**Figure 7A**), and ORP showed no significant differences except that M3 had the highest ORP (**Figure 7D**), consistent with higher DO and aerobic conditions.

**Figure 7:**
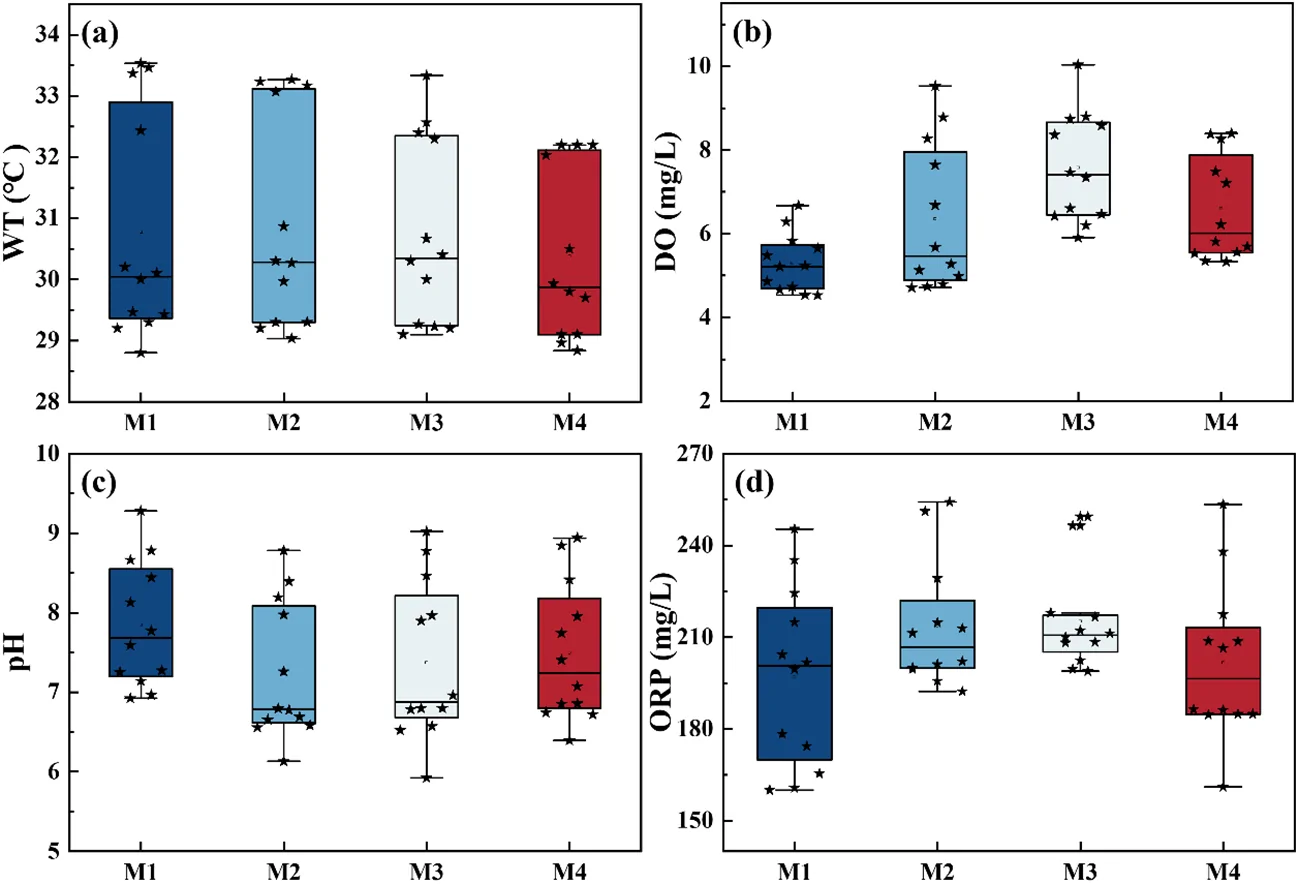
Differences in WT **(A)**, DO **(B)**, pH **(C)** and ORP **(D)** in water under different planting substrates.

For phosphorus (**Supplementary Figure 3**), M3 achieved the highest removal loads of TP (2.70×10^-4^ g·m^-^²·d^-^¹) and phosphate (2.40×10^-4^), which were 2.70- and 2.40-fold those of M1, respectively. For nitrogen (**Figure 8**), M3 had the highest removal loads of TN (2.32×10^-4^ g·m^-^²·d^-^¹) and NO_3_^-^-N (1.35×10^-4^ g·m^-^²·d^-^¹), but the lowest NH_4_^+^-N removal load (0.87×10^-4^ g·m^-^²·d^-^¹). This pattern suggests that the relatively dense fiber network of the linen substrate may create localized anaerobic microenvironment that favor denitrification (converting NO_3_^-^ to N_2_) while potentially suppressing nitrification (converting NH_4_^+^ to NO_3_^-^), as nitrifiers require oxygen. This pattern provides a plausible mechanistic link between substrate structure and nitrogen transformation pathways.

**Figure 8:**
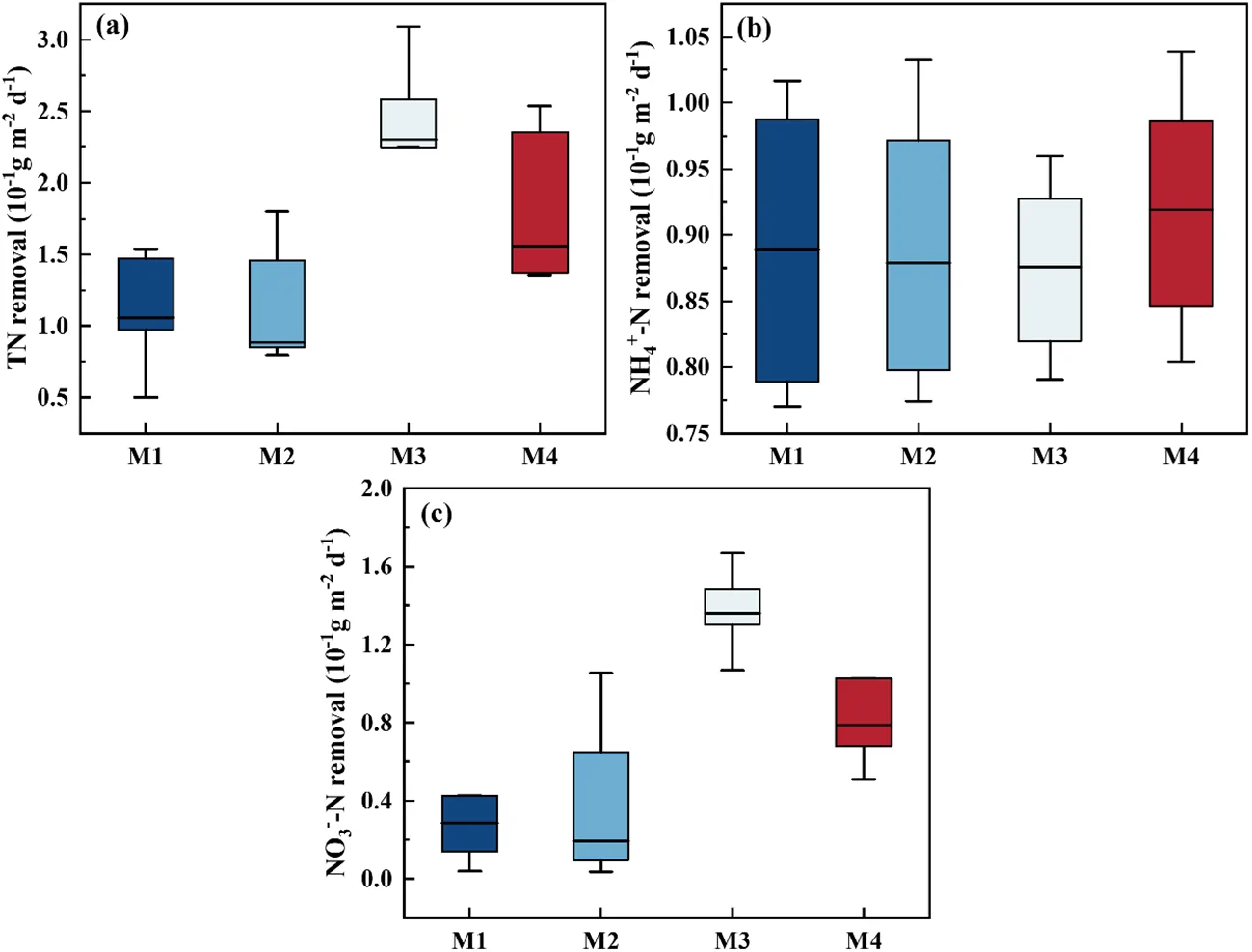
Removal loads of total nitrogen **(A)**, ammonia nitrogen **(B)**, and nitrate nitrogen **(C)** in water bodies under different planting substrates.

### 3.3 Surface microstructure of substrates

SEM images (**Figure 9**) revealed distinct morphological features. M1 (plastic net) had a smooth, regular surface with a thin, uniform particle layer prone to cracking. M2 (palm mat) consisted of interwoven natural fibers with rough surfaces, heavily covered by particle aggregates. M3 (linen cloth) displayed a complex, three-dimensional network of fine fibers with numerous interstitial spaces, which were extensively filled with particles, suggesting a high capacity for particle retention and potential biofilm development. M4 (bamboo strips) had a relatively flat surface with longitudinal depressions, only partially covered by particles. These microstructural differences are consistent with the superior turbidity and nutrient removal performance observed in M3, likely because the high porosity and surface area of linen promote particle retention and biofilm establishment.

**Figure 9:**
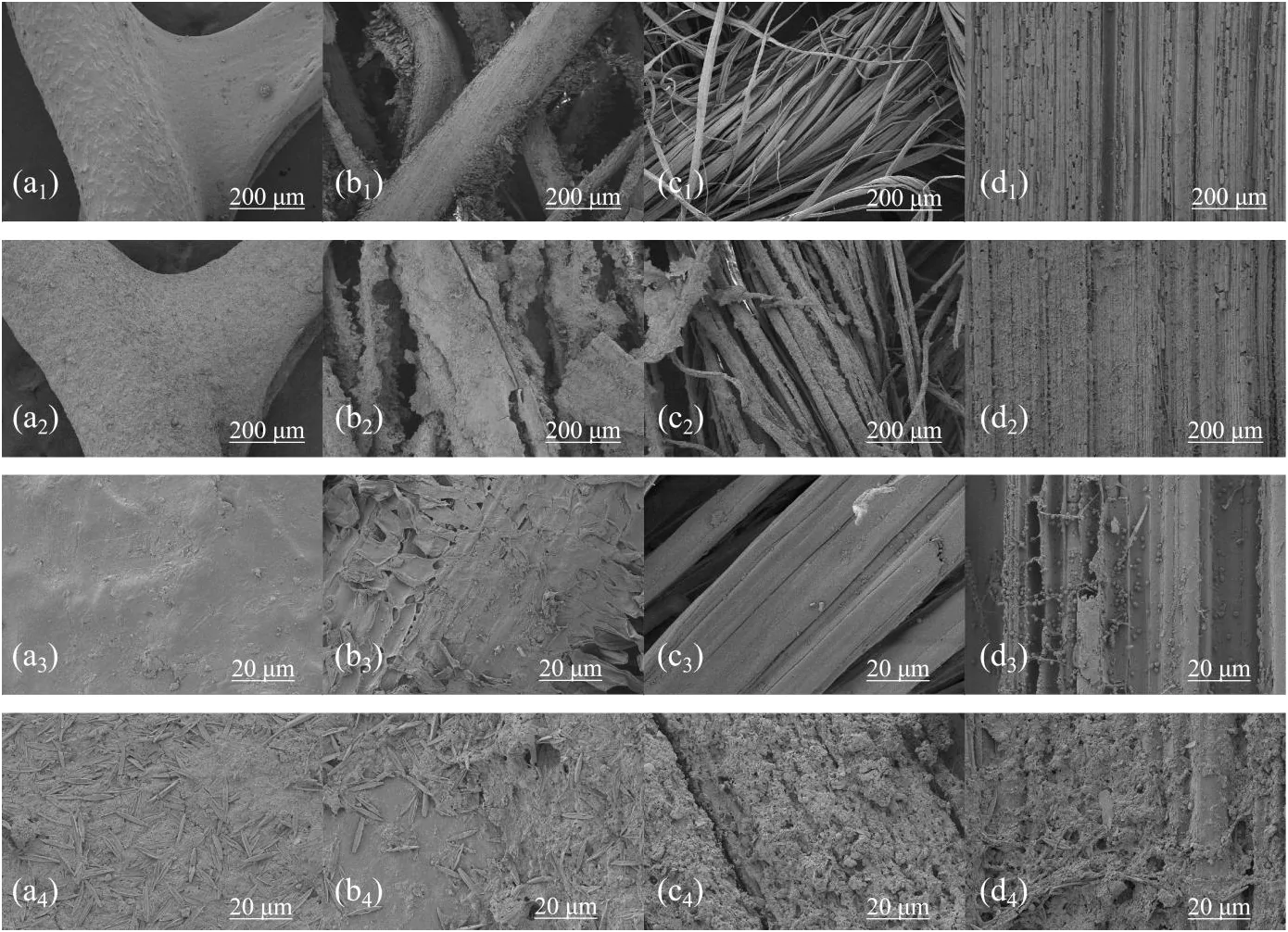
SEM image of the planting substrate surface (Letters a to d in the figure represent the planting substrates M1 to M4 in sequence, and the numbers 1 and 2 represent the SEM images before and after the experiment at 200 μm, respectively; the numbers 3 and 4 represent the SEM images before and after the experiment at 20 μm, respectively).

### 3.4 Microbial community structure

#### 3.4.1 Alpha diversity and taxonomic composition

Alpha diversity indices (Shannon, Simpson, Ace, Chao) are summarized in **Figure 10**. Across all samples, root surfaces (R) exhibited the highest species richness and diversity, followed by leaf surfaces, substrate surfaces, and water column. Over time, diversity increased in most compartments. Notably, the Ace index on root surfaces fluctuated within 668–1102, while on substrate surfaces it was 456–884, suggesting that the rhizosphere served as a primary hotspot for microbial colonization and community stability.

**Figure 10:**
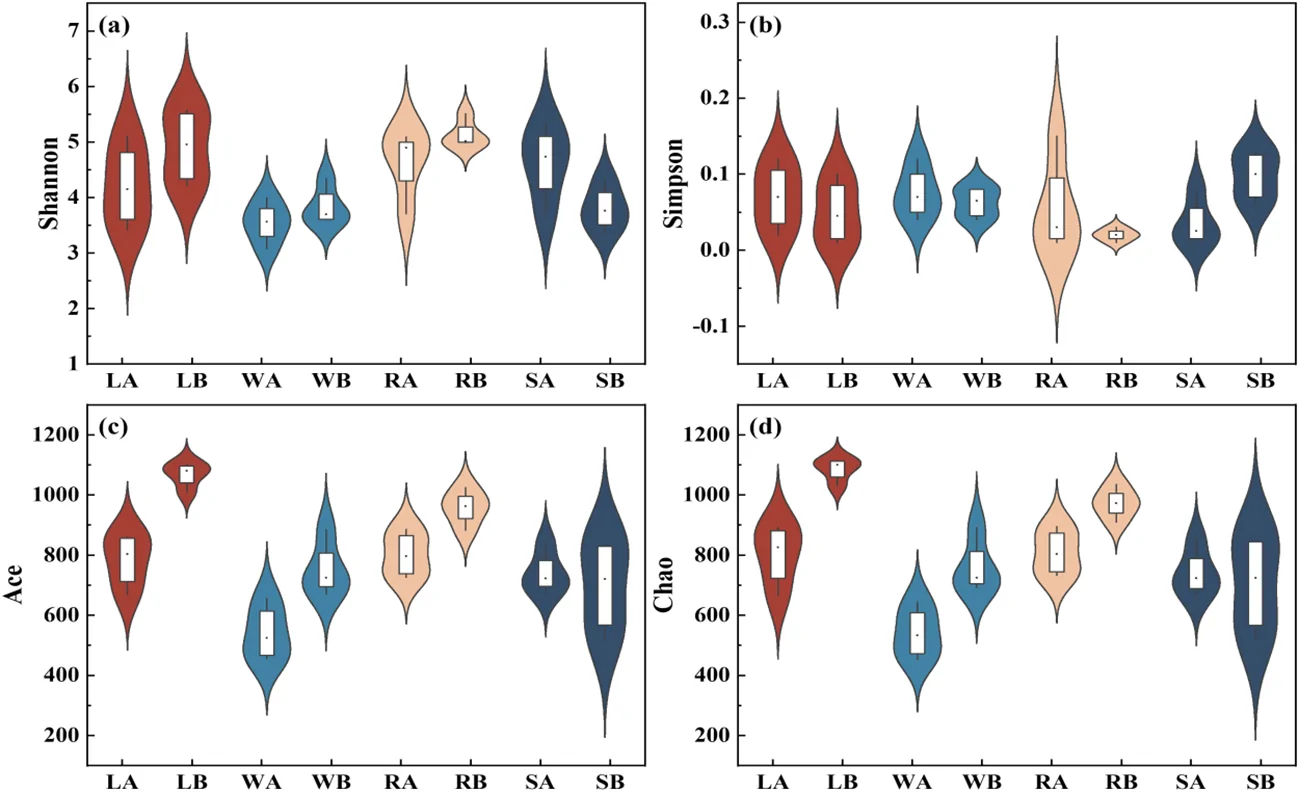
Microbial diversity indices of sampling areas: **(a)** Shannon, **(b)** Simpson, **(c)** Ace, **(d)** Chao (OTU level). Sample codes: L, leaf surface; R, root surface; S, substrate surface; W, water column; A, mid-stage (day 21); B, late-stage (day 42); (e.g., LA = mid-stage leaf sample).

**Supplementary Table 2** lists the number of taxa at each taxonomic level. At the genus level, late-stage leaf samples had the highest count (319), followed by roots (217), water (153), and substrates (103). This temporal and spatial variation suggests that substrate type differentially selects for specific microbial consortia.

#### 3.4.2 Dominant phyla and genera

At the phylum level (**Figure 11**), *Proteobacteria*, *Actinobacteriota*, *Bacteroidota*, *Patescibacteria*, *Acidobacteriota*, *Chloroflexi*, and *Deinococcota* were dominant (>70% relative abundance) across all samples, with minor variations among treatments.

**Figure 11:**
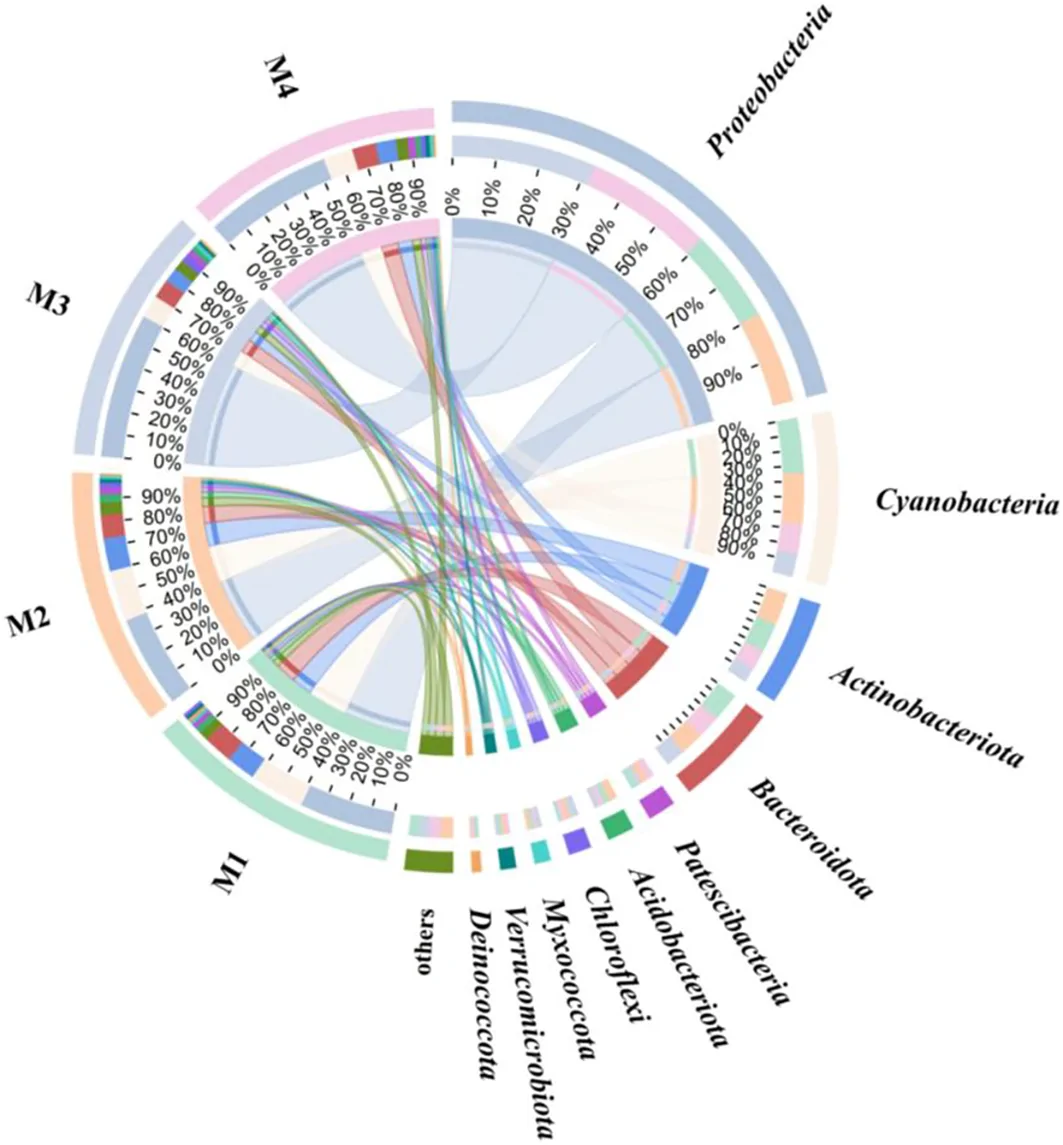
Relative abundance of dominant microbial groups under different planting substrates (phylum level).

At the genus level (**Figure 12**; **Supplementary Figure 4**), *Comamonadaceae*—a family encompassing members with known denitrification capacity (Liang et al., 2022)—was widely present on leaf (**Figure 12A**) and root surfaces, with the highest relative abundance on the root surface of M3 in the late stage (**Figure 12B**). This higher abundance in M3 coincided with the elevated TN and NO_3_^-^ removal loads observed in this treatment. Other notable genera included *Aeromonas*, *Ideonella*, and *Rhodobacter*, with distinct temporal dynamics. In water samples (**Supplementary Figure 4A**), M3 enriched *Paenarthrobacter* (a plant growth-promoting genus producing indole-3-acetic acid) and *Polynucleobacter* (typical of oligotrophic waters), consistent with better plant growth and lower nutrient levels (Deutch et al., 2018; Zhang et al., 2024; Wu and Hahn, 2006). On substrate surfaces (**Supplementary Figure 4B**), M3 showed a marked increase in *Rhodoblastus* (photosynthetic denitrifier) from 10.71% to 28.74% over time, replacing *Phaeospirillum* as the dominant genus.

**Figure 12:**
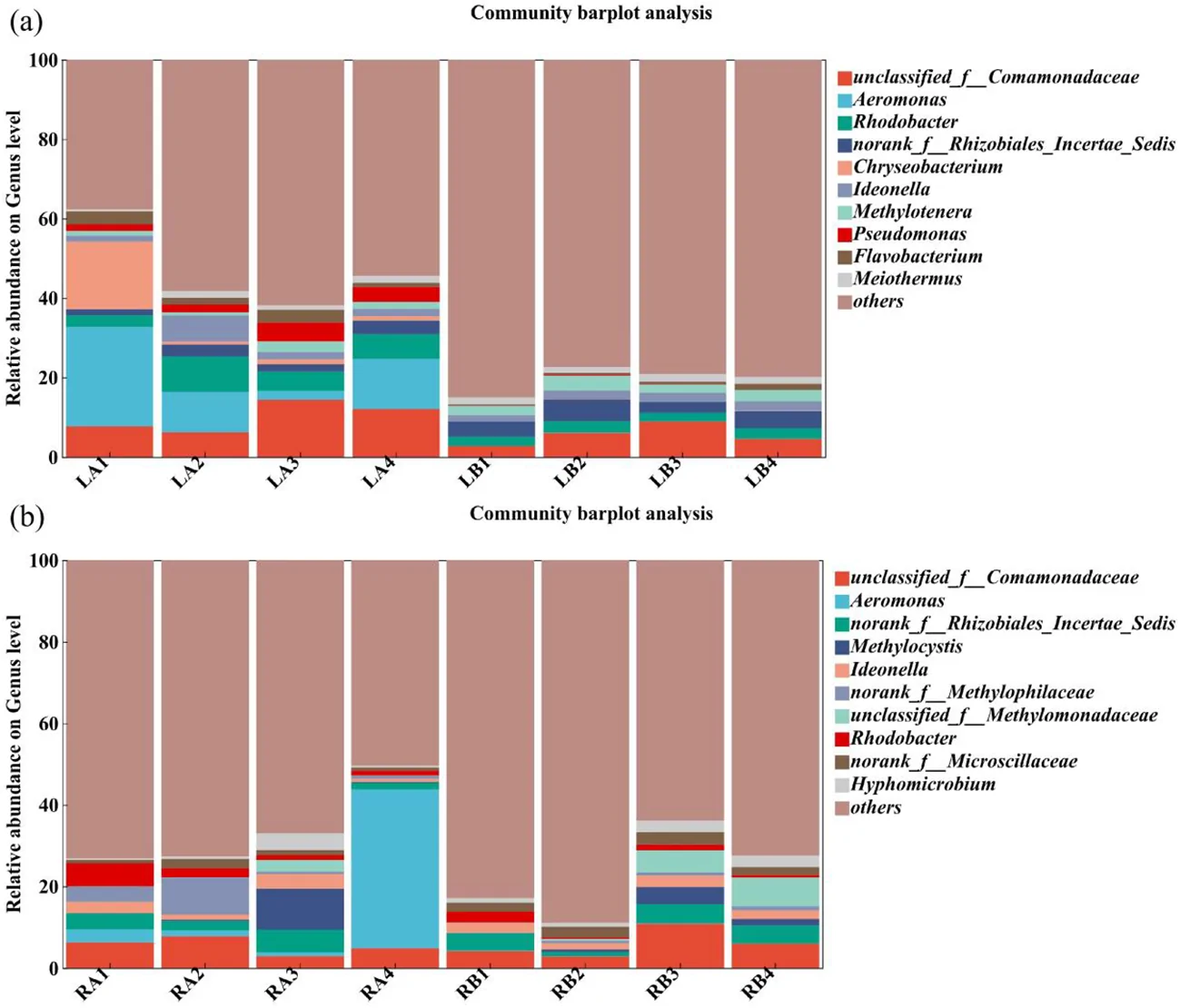
Relative abundance of microbial communities on the leave surface **(A)** and root system **(B)** of *V. spinulosa* Yan (genus level).

#### 3.4.3 Community differentiation and functional indicators

PLS-DA (**Figure 13**) demonstrated that M3 and M4 formed a tight cluster, distinct from M1 and M2, suggesting substrate-specific selection of microbial communities. Compartment-wise, leaf (L) and water (W) communities were separated, while root (R) and substrate (S) communities overlapped, suggesting close interaction between rhizosphere and substrate biofilms.

**Figure 13:**
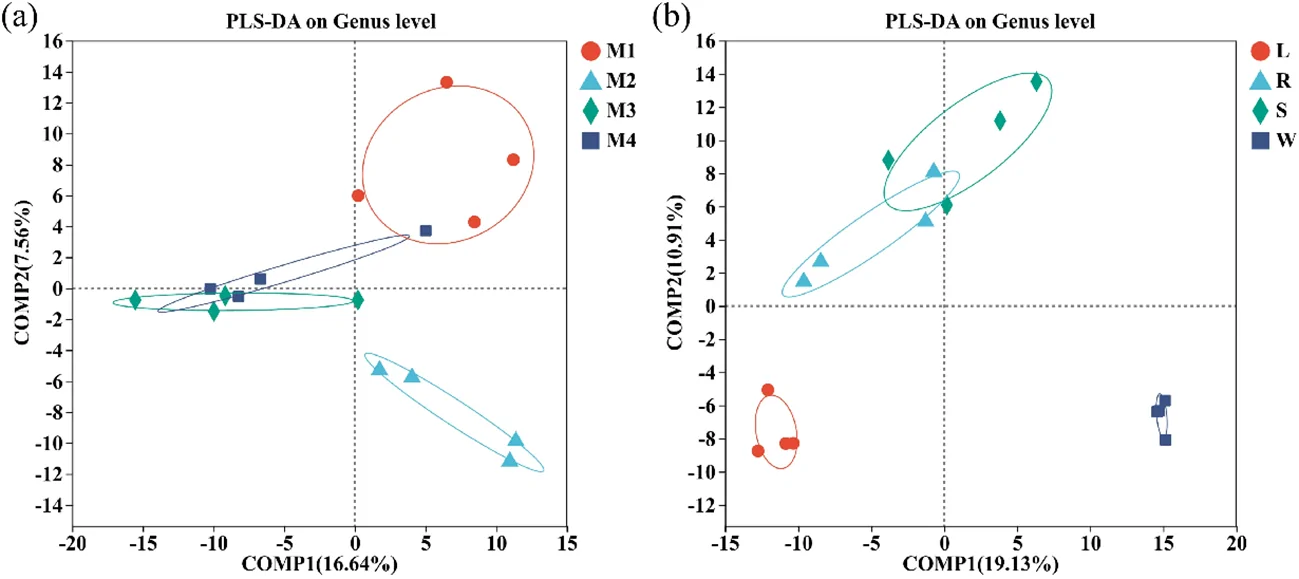
PLS-DA analysis of microbial communities for different planting substrates **(A)** and different sampling areas **(B)** (genus level).

Differential abundance analysis (**Figure 14**) identified *Sphingomonadaceae* (enriched in M1, possibly degrading polypropylene), *Rhizomicrobium* and *Sporomusaceae* (only present in M3 and M4, known to participate in denitrification or provide substrates for denitrifiers) (Thelusmond et al., 2016; Han et al., 2019; Jia et al., 2022). These taxa likely contribute to the superior nitrogen removal observed in M3 and M4.

**Figure 14:**
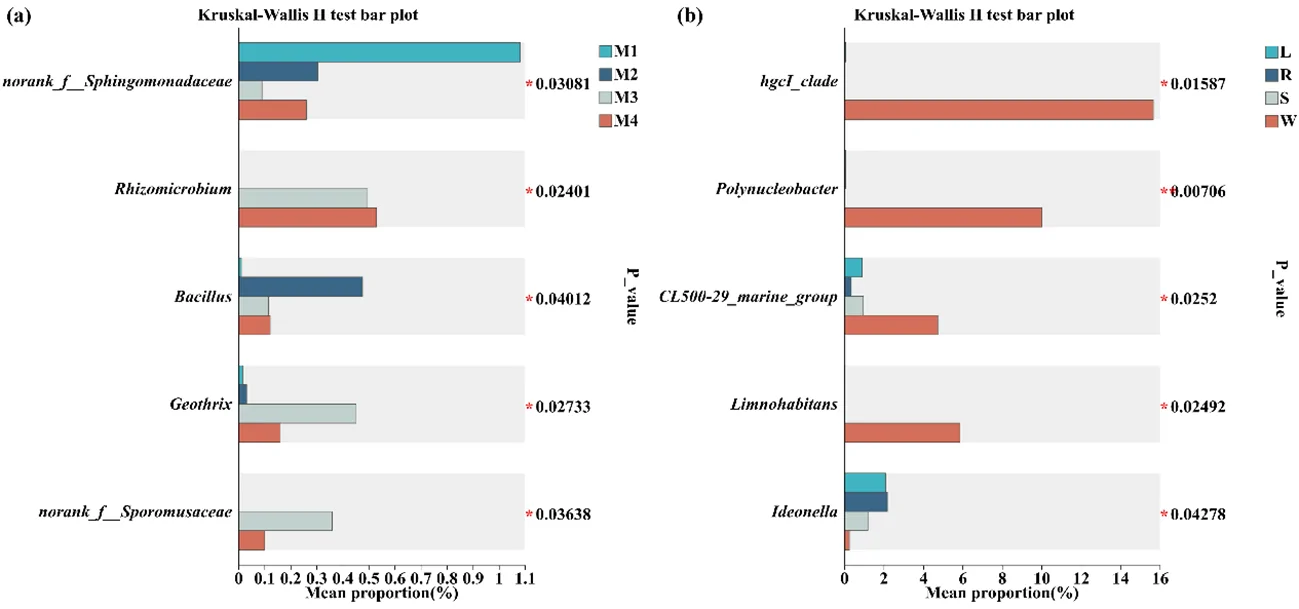
Analysis of microbial community differences among different planting substrates **(A)** and different sampling areas **(B)** (genus level).

In summary, the microbial data suggest that linen cloth was associated with higher diversity and more complex community structures, as well as enrichment of denitrifying and plant-growth-promoting bacteria, which may collectively contribute to a synergistic plant–microbe system that supports stress tolerance and water purification.

## 4 Discussion

This study presents multiple lines of evidence suggesting that the choice of planting substrate in suspended *V. spinulosa* Yan systems is associated with differences in plant oxidative stress status, antioxidant defense capacity, rhizosphere microbial community assembly, and ultimately the resilience of the entire plant–microbe symbiotic system under high-turbidity stress. Among the four tested substrates, linen cloth (M3) showed the most favorable performance among the four treatments, with the greatest measured physiological benefits and functional improvements. These findings contribute to the understanding of redox regulation in aquatic plant–microbe symbioses and suggest potential mechanistic directions for ecological restoration engineering.

### 4.1 Substrate physical structure determines root development and oxidative stress alleviation

The superior growth of *V. spinulosa* in the linen cloth treatment may be related to its unique porous, fibrous architecture. Unlike the smooth, large-mesh plastic net (M1) or the overly dense palm mat (M2) and rigid bamboo strips (M4), linen cloth provides a loose yet stable matrix that allows roots to penetrate, anchor, and expand freely. This physical facilitation may enhance nutrient and water uptake, potentially supporting aboveground biomass accumulation and photosynthetic pigment synthesis. The results of this study align with previous studies, emphasizing that substrate physical and chemical properties critically influence submerged macrophyte growth (Barko et al., 1988; Li et al., 2012). Specifically, higher substrate bulk density can impose physical impedance on root penetration and anchorage, as demonstrated by Barko and Smart (Barko and Smart, 1986) and Barko et al (Barko et al., 1991), who showed that sediment density above a threshold restricts root elongation in submersed macrophytes. However, our data provide additional observations linking substrate structure to oxidative stress physiology.

Under high turbidity, light limitation inevitably leads to an imbalance between photosynthetic electron transport and carbon fixation, resulting in ROS overproduction. Previous studies have confirmed that light attenuation in turbid waters can induce oxidative stress in submerged macrophytes. For example, Zhang et al. (2010) found that exposure to reduced sunlight (42% and 11% of full irradiance) enhanced the activities of superoxide dismutase and guaiacol peroxidase in *Potamogeton crispus*, coinciding with altered carbon and nitrogen metabolism. Zhu et al. (2024) further demonstrated that, under shaded conditions, catalase activity exhibited a pronounced increase while other antioxidant enzymes declined, suggesting that catalase plays a predominant role in the antioxidative response of *Potamogeton crispus* to low-light stress. Plants cope with this via enzymatic antioxidants (SOD, POD, CAT) that scavenge ROS, and MDA serve as a reliable indicator of membrane damage. In M3, the lowest MDA and the most stable, moderate increases in SOD/POD/CAT activities are consistent with the interpretation that linen cloth may have reduced the initial ROS burden and enabled a relatively well-regulated antioxidant response. In contrast, M1 exhibited continuously rising MDA and sharp late-stage increases in SOD, suggesting chronic stress and a compensatory, less efficient defense. This differential response may involve two non-mutually exclusive mechanisms: (1) the well-developed root system in M3 may enhance overall plant vigor and stress tolerance; and (2) the linen substrate’s high adsorption capacity may rapidly reduce turbidity, improving light penetration and alleviating the primary trigger of ROS production. Min et al. (2017) observed a similar buffered antioxidant response in *V. natans* under moderate nitrate levels, which is consistent with the notion that substrate-mediated environmental amelioration reduces oxidative pressure.

### 4.2 Substrate-mediated microenvironment shapes nitrogen transformation and water purification

The distinct nutrient removal patterns—high TN and NO_3_^-^ removal but low NH_4_^+^ removal in M3—suggest a potential role of substrate-induced redox microenvironments. Linen cloth, with its dense fiber network and high water retention, tends to create local anaerobic or anoxic zones within the substrate and near the root surface after water saturation. Such conditions may favor denitrifying bacteria that reduce NO_3_^-^ to N_2_, while potentially suppressing aerobic nitrifiers that oxidize NH_4_^+^ to NO_3_^-^. This is consistent with the enrichment of *Comamonadaceae* and *Rhizomicrobium* on M3 roots and substrate surfaces—both known denitrifiers (Liang et al., 2022; Wang et al., 2018). The relatively low NH_4_^+^ removal in M3 may reflect a greater contribution of denitrification relative to nitrification, a possibility suggested by the enrichment of known denitrifiers but not directly verified by rate measurements in this study. In contrast, M4 (bamboo) with a looser structure allowed better oxygen exchange, achieving a balance between nitrification and denitrification, resulting in moderate removal of both NH_4_^+^ and NO_3_^-^. This substrate-dependent pattern of nitrogen transformations is qualitatively similar to observations from Wang et al. (2018) and Deng et al. (2024), who reported that macrophytes shape rhizosphere microbial functions via oxygen and exudate release. This study raises the possibility that substrate materials themselves may impose additional redox gradients that influence these microbial processes.

For phosphorus, the high removal in M3 may involve combined plant uptake, substrate adsorption (high specific surface area), and microbial assimilation by polyphosphate-accumulating organisms, which are likely enriched in the complex biofilm on linen fibers. This is consistent with previous observations that submerged macrophytes can significantly enhance phosphorus removal through both direct uptake and by providing surfaces for phosphorus-accumulating biofilms. Substrate properties such as porosity and surface roughness have been shown to influence microbial colonization and subsequent phosphorus immobilization. Furthermore, the rhizosphere microbial communities associated with submerged macrophytes play a critical role in phosphorus cycling by mediating the transformation between different phosphorus fractions (Pan et al., 2025). Notably, phosphorus availability has also been shown to enhance the stress resistance and adaptability of *Vallisneria natans* under heavy metal exposure (Pb²^+^ and Cd²^+^), with biofilms developing more abundant and diverse microbial communities under combined phosphorus and metal stress (Huang S. et al., 2022). This observation suggests that phosphorus not only serves as a nutrient but also plays a protective role in plant–microbe symbiotic systems under co-stressor conditions.

### 4.3 Rhizosphere microbiome as a bridge between plant health and ecosystem function

The microbial community analysis suggests that substrate selection is associated with differences not only in plant physiology but also in the assembly of the rhizosphere microbiome. The root surface harbored the highest diversity and richness, confirming the rhizosphere as a hotspot of plant–microbe interactions. The enrichment of *Paenarthrobacter* in M3 water is consistent with a positive feedback loop: reduced oxidative stress may promote root exudation, which in turn could support beneficial bacteria that enhance nutrient acquisition and stress tolerance (Salimi et al., 2023). This positive feedback between plant physiological status and rhizosphere microbial functionality has been increasingly recognized in aquatic plant systems. Liu et al. (2024) investigated how leaf damage in submerged macrophytes affects rhizosphere conditions, revealing that the release of organic acids modifies the composition and abundance of denitrifying bacterial communities, with differential responses observed between denitrifiers and anammox bacteria. Moreover, the diversity and composition of rhizosphere bacterial communities are closely linked to plant health and stress tolerance, with healthier plants generally supporting more diverse and functionally redundant microbial assemblages. Similarly, the dominance of *Polynucleobacter* in M3 water is consistent with oligotrophic conditions, which aligns with the low nutrient levels measured in this treatment.

The PLS-DA clustering of M3 and M4, and their separation from M1 and M2, suggest that substrate type may exert selective pressure on microbial community composition. This observation is consistent with findings from other studies on submerged macrophyte restoration, where different substrate amendments were shown to significantly alter rhizosphere microbial community structure and promote the growth of functional microorganisms. The selective enrichment of specific microbial taxa by substrate type likely arises from differences in surface chemistry, porosity, and nutrient availability, which together create distinct microhabitats for microbial colonization. Studies have shown that combined application of substrate materials (e.g., Ca/Al composites) with submerged macrophytes not only facilitates sediment phosphorus immobilization but also substantially modifies the composition and functional potential of microbial communities associated with both sediments and leaf biofilms, thereby optimizing nitrogen and phosphorus cycling functions in the rhizosphere (Zhang et al., 2025). Furthermore, Yin et al. (2020) further showed that organic acids exuded from macrophyte roots—particularly citric and oxalic acids—serve as key environmental drivers shaping the abundance of *nirS*-type denitrifiers and anammox bacteria in the rhizosphere. The presence of *Rhizomicrobium* and *Sporomusaceae* exclusively in M3 and M4—both linked to denitrification—is consistent with the notion that natural fibrous substrates may provide more favorable niches for nitrogen-cycling microbes than synthetic plastic or compressed palm. Moreover, the high abundance of *Rhodoblastus* on M3 substrate surfaces, a photosynthetic bacterium capable of anaerobic anoxygenic photosynthesis, suggests that the light-transmitting and anaerobic microenvironment within linen fibers may support unique metabolic guilds that contribute to nitrogen and carbon cycling.

Crucially, the complex microbial network inferred from diversity and co-occurrence patterns (though not directly calculated in this study, the higher diversity and coexistence of multiple functional groups) in M3 suggest a potentially more resilient microbial assemblage. The concept of microbial network resilience has been increasingly applied to understand the stability of plant–microbe symbiotic systems under environmental stress. Hao et al. (2025) documented that increasing eutrophication stress drives a functional transition in endophytic bacterial assemblages associated with submerged macrophytes—from nutrient-procuring traits toward stress-counteracting strategies—concurrently with reduced complexity and stability of the microbial co-occurrence network. Similarly, Pan et al. (2025) further observed that macrophytes exhibiting higher phosphorus stoichiometric homeostasis supported richer bacterial diversity; network analysis indicated that high-homeostasis systems contained 91% more microbial nodes and 105% more positive inter-taxa associations relative to their low-homeostasis counterparts. Studies have also shown that submerged macrophytes significantly increase the population of nitrogen-converting bacteria and enhance the expression of nitrification and denitrification genes, highlighting the critical role of rhizosphere microbial communities in maintaining ecosystem functions. This resilience—the capacity to maintain functions under environmental perturbation—is a key property of symbiotic systems facing oxidative challenges. The positive correlations among reduced oxidative stress (low MDA), high antioxidant enzyme activities, and the enrichment of denitrifiers and PGPB suggest an association between plant redox status and microbiome functionality. One possible mechanism for this association is through root exudation: reduced stressed may allow greater carbon to exudates, potentially supporting a diverse microbiome that could reciprocally benefits the plant through nutrient mobilization and stress alleviation.

### 4.4 Implications for ecological restoration and future perspective

Among the substrates tested, linen cloth showed the most promising performance for suspended planting of *V. spinulosa* Yan in turbid waters, not only as a physical carrier but potentially also as a biological active interface associated with reduced oxidative stress, enhanced antioxidant defenses, and a more complex rhizosphere microbiome. From a practical perspective, the successful application of this technology in the field requires careful attention to two engineering considerations: (1) the substrate mat must be secured with appropriate counterweights to overcome buoyancy and maintain the planting plane at the target depth; and (2) the suspension depth should be adjustable to accommodate seasonal variations in water transparency and plant growth stage. These requirements can be readily met using modular suspended planting units with weighted frames and adjustable ropes—a design that has been successfully implemented in various field-scale restoration projects for submerged macrophytes in turbid or deep-water environments. The present study provides the substrate-selection basis for such engineering applications, with linen cloth identified as the optimal material among the four candidates tested. This integrated perspective is consistent with emerging evidence that successful restoration of submerged macrophytes depends not only on plant establishment but also on the coordinated recovery of rhizosphere microbial communities and their associated ecosystem functions (Wang et al., 2018; Deng et al., 2024). This integrated perspective moves beyond traditional engineering assessments and aligns with the emerging paradigm that successful restoration hinges on managing plant–microbe symbioses under stress.

Several limitations of this study should be acknowledged. First, although the outdoor flume experiment was conducted under controlled conditions with three technical replicates per sampling event, it cannot fully capture the environmental complexity of natural water bodies—such as fluctuating hydrology, multiple coexisting pollutants, and seasonal variations. Moreover, only one experimental unit was established per substrate treatment. Field-scale validation with true biological replication over extended timescales is therefore necessary to confirm the reproducibility and long-term efficacy of our findings. Consequently, all statistical comparisons reported in this study are descriptive in nature and should not be interpreted as definitive evidence of treatment effects. The observed consistent trends across multiple independent physiological, water quality, and microbial indicators lend internal coherence to our conclusions, but independent biological replication is essential for statistical generalization. Field-scale validation over extended timescales is therefore necessary to confirm the long-term efficacy and stability of the linen-based suspended planting system. Second, while our 16S rRNA sequencing revealed shifts in microbial community structure and taxonomic composition, they do not directly quantify the expression of stress-response genes or nitrogen-metabolism pathways *in situ*. Moreover, the PICRUSt2-based functional predictions presented in this study are computational inferences derived from 16S rRNA gene sequences and reference genome databases, they do not represent direct measurements of gene transcription or enzyme activity. Therefore, the inferred functional potential should be interpreted as a hypothesis-generating tool rather than as definitive evidence of *in situ* microbial functions. Furthermore, the specific chemical components of linen (e.g., cellulose, lignin) that might serve as slow-release carbon sources for denitrifiers remain unexplored. Future investigations employing metagenomics, metatranscriptomics, or targeted qPCR—coupled with chemical characterization of substrate degradation products—will be essential to elucidate the molecular and chemical mechanisms underpinning the observed improvements in plant antioxidant defense and microbial nitrogen removal.

## 5 Conclusion

This study demonstrates that substrate selection in suspended *V. spinulosa* Yan systems critically influences plant oxidative stress status, antioxidant defense capacity, rhizosphere microbial community structure, and water purification performance under high-turbidity stress. Among the four substrates tested, linen cloth proved superior across all evaluated aspects: it promoted root development and biomass accumulation, increased photosynthetic pigment content, and most importantly, alleviated oxidative damage (lowest MDA) while maintaining well-regulated SOD, POD, and CAT activities. Mechanistically, the dense porous network of linen cloth creates anaerobic microenvironments that favor denitrifying bacteria (e.g., *Comamonadaceae*, *Rhizomicrobium*), thereby enhancing nitrate removal, while simultaneously supporting plant-growth-promoting genera (*Paenarthrobacter*) and oligotrophic indicators (*Polynucleobacter*). These results establish a mechanistic framework linking substrate physical properties to plant redox regulation and microbiome-mediated ecosystem functions in suspended planting systems.

For practical application, linen cloth represents a promising candidate substrate for turbid-water restoration, yet several challenges remain. Field validation under diverse hydrological and climatic conditions is essential to confirm the reproducibility and long-term stability of the observed benefits. The molecular mechanisms underlying substrate-mediated ROS signaling and antioxidant regulation require further elucidation through metagenomic and metatranscriptomic approaches. Additionally, the degradation kinetics and environmental fate of linen substrates in natural water bodies merit systematic investigation to assess their lifespan and potential secondary effects. Addressing these knowledge gaps through integrated field and laboratory studies will be critical for translating this proof-of-concept finding into scalable, cost-effective restoration practice.

## Data Availability

The datasets presented in this study can be found in online repositories. The names of the repository/repositories and accession number(s) can be found below: https://www.ncbi.nlm.nih.gov/, PRJNA1520848.
