# Supplementary material for: Substrate-mediated oxidative stress alleviation and rhizosphere microbiome modulation enable submerged macrophyte restoration in high-turbidity water
Source: Front Plant Sci. 2026 Oct 30;17:1961849. doi: 10.3389/fpls.2026.1961849 (PMC13759781; doi:10.3389/fpls.2026.1961849)
Supplement: Supplementary file 1 [file Table1.docx]

Supplementary Material

for

**Substrate-mediated oxidative stress alleviation and rhizosphere microbiome modulation enable submerged macrophyte restoration in high-turbidity water**

**Supplementary Figure 1.** Schematic diagram of the suspended planting facility. (1: PVC barrel (experimental tank); 2: Y-shaped stainless steel support frame; 3: Horizontal suspension rod; 4: Manual hand winch for depth adjustment; 5: Nylon suspension ropes; 6: Planting platform with substrate layer and submerged macrophytes (*Vallisneria spinulosa* Yan); 7: Counterweight ball for balance maintenance. The planting plane is suspended at 20 cm below the water surface and can be vertically adjusted via the hand winch)

**Supplementary Figure 2.** Particle size of suspended solids attached to leaves under different planting substrates: (a) M1 group, (b) M2 group, (c) M3 group, (d) M4 group.

**Supplementary Figure 3.** Total phosphorus (a) and phosphate (b) removal loads in water under different planting substrates.

**Supplementary Figure 4.** Relative abundance of microbial communities in the water body (a) and on the planting substrate surface (b) (genus level).

**Supplementary Table 1**

Definitions of combined sample codes

| Combined code | Compartment | Sampling time | Full description |
| --- | --- | --- | --- |
| LA | Leaf | Day 21 (mid‑experiment) | Leaf samples collected at mid‑experiment (without treatment specification) |
| LB | Leaf | Day 42 (late‑experiment) | Leaf samples collected at late‑experiment (without treatment specification) |
| RA | Root | Day 21 (mid‑experiment) | Root samples collected at mid‑experiment (without treatment specification) |
| RB | Root | Day 42 (late‑experiment) | Root samples collected at late‑experiment (without treatment specification) |
| SA | Substrate | Day 21 (mid‑experiment) | Substrate samples collected at mid‑experiment (without treatment specification) |
| SB | Substrate | Day 42 (late‑experiment) | Substrate samples collected at late‑experiment (without treatment specification) |
| WA | Water | Day 21 (mid‑experiment) | Water samples collected at mid‑experiment (without treatment specification) |
| WB | Water | Day 42 (late‑experiment) | Water samples collected at late‑experiment (without treatment specification) |

**Supplementary Table 2**

Statistics of microbial classification levels.

| **Sample Type** | **Phylum** | **Class** | **Order** | **Family** | **Genus** | **Species** |
| --- | --- | --- | --- | --- | --- | --- |
| Mid-stage Leaf (LA) | 19 | 35 | 85 | 128 | 206 | 271 |
| Mid-stage Water (WA) | 10 | 16 | 45 | 65 | 119 | 153 |
| Mid-stage Root (RA) | 19 | 41 | 102 | 153 | 244 | 316 |
| Mid-stage Substrate (SA) | 20 | 34 | 79 | 116 | 182 | 316 |
| Late-stage Leaf (LB) | 27 | 58 | 138 | 203 | 319 | 441 |
| Late-stage Water (WB) | 17 | 30 | 68 | 102 | 153 | 183 |
| Late-stage Root (RB) | 21 | 38 | 99 | 142 | 217 | 282 |
| Late-stage Substrate (SB) | 17 | 29 | 557 | 76 | 103 | 125 |
